# The complete chloroplast genome of *Photinia davidsoniae*: molecular structures and comparative analysis

**DOI:** 10.1080/23802359.2021.1911698

**Published:** 2021-04-19

**Authors:** Jingling Li, Mei Jiang, Liqiang Wang, Jie Yu, Haimei Chen, Jingting Liu, Chang Liu

**Affiliations:** a College of Horticulture and Landscape Architecture, Southwest University, Chongqing, China; b Key Laboratory of Bioactive Substances and Resource Utilization of Chinese Herbal Medicine from Ministry of Education, Engineering Research Center of Chinese Medicine Resources from Ministry of Education, Institute of Medicinal Plant Development, Chinese Academy of Medical Sciences, Peking Union Medical College, Beijing, China; c Key Laboratory of Horticulture Science for Southern Mountainous Regions, Ministry of Education, Chongqing, China

**Keywords:** *Photinia*, chloroplast, plastome, comparative analysis, phylogenetic analysis

## Abstract

*Photinia davidsoniae* is a common ornamental arbor in the genus *Photinia* (family Rosaceae). Here, we sequenced and assembled the complete plastome of *P. davidsoniae* using the next-generation DNA sequencing technology. And we then compared it with nine *Photinia* species using a range of bioinformatics software tools. The ten plastomes had sizes ranging from 159,230 bp for *P. beckii* to 160,346 bp for *P. davidsoniae*. They all had a conservative quartile structure. It contained two single-copy regions: a large single-copy (LSC) region, a small single-copy (SSC) region, and a pair of inverted repeat (IR) regions. Each of the plastomes encoded 113 unique genes, including 79 protein-coding genes, four rRNA genes, and 30 tRNA genes. Furthermore, we detected six hypervariable regions (*mat*K-*rps*16, *rpo*B-*trn*C, *trn*T-*psb*D, *ndh*C-*trn*V, *psb*E-*pet*L, *ndh*F-*rpl*32-*trn*L), which could be used as potential molecular markers. We constructed two phylogenetic trees with plastomes or concatenated protein sequences of 25 species of 8 genera of Rosaceae. The tree constructed with complete plastomes has much stronger support. The results placed *P. davidsoniae* in the upper part of the phylogenetic tree. It shows that *P. davidsoniae* and *P. lanuginosa* are closely related. In summary, the plastomes of *Photinia* are conserved overall but carry significant minor variations, as expected. The results will be indispensable for distinguishing species, understanding the interspecific diversity, and elucidating the evolutionary processes of *Photinia* species.

## 1. Introduction

The genus *Photinia* belongs to Maleae (Rosaceae) and comprises approximately 60 species (Robertson et al. 1991; Lu and Spongberg 2003). They are widespread landscape tree species resistant to pruning and air pollution (Mattei et al. 2017; Mori et al. 2018), and many were cultivated for gardening (Zhao et al. 2020). *Photinia davidsoniae* Rehder & E.H.Wilson (referred to as *P. davidsoniae* in the following text) is an evergreen plant species, which grows in thickets at altitudes of 600–1000 m and mainly distributes in southern China and Southeast Asia (Lu and Spongberg 2003). Like many other species of *Photinia*, *P. davidsoniae* has luxuriant foliage around the trunk, purple and tender leaves in early spring, and little white flowers in early summer, and bear red fruits in autumn. (Sterling 1965; Aoki et al. 2006; Mattei et al. 2018). *Photinia* species exhibited similar morphological features and the species boundaries have been unclear. With the continuous discovery of new *Photinia* species (Guo et al. 2010; Li et al. 2015), a reliable classification of *Photinia* is in urgent need.

Chloroplast genomes (referred to as plastomes in the following text) have been widely used in plant taxonomy. Compared with morphological identification, plastome sequences can produce more accurate phylogenetic relationships. Recently, the confusion in the taxonomic of *Photinia*-related species has been primarily solved based on the complete plastome sequences (Shi et al. 2019; Liu, Liu, et al. 2019). Particularly, the phylogenetic analysis of the *Photinia*-related species support the idea of a new genus *Phippsiomeles* and the resurrection of a redefined *Stranvaesia* in Maleae (Liu, Hong, et al. 2019). Furthermore, *Eriobotrya* was found to belong to *Rhaphiolepis* based on plastomes and ribosome DNA (Liu, Liu, et al. 2019). However, their study focused only on the phylogenetic relationships among *Photinia* and its related genera. The comparative analysis of Photinia plastomes were not conducted extensively.

Here, we sequenced and assembled the complete plastomes of *P*. *davidsoniae* for the first time and then compared them with the plastomes of nine published *Photinia* species to explore the interspecific diversity of the plastomes of *Photinia*.

## 2. Materials and methods

### 2.1. Plant material, DNA extraction, and sequencing

We collected fresh leaves of *P. davidsoniae* from the Central China Medicinal Botanical Garden, EnShi, China (30°10′N, 109°44′E) and froze them at −80 °C. We used the plant genomic DNA kit (Tiangen Biotech, Beijing) to extract the total DNA following the manufacturer's protocol (Zhang, Li, et al. 2019). The DNA library with an insert size of 350 bp was constructed using the library preparation kit (New England Biolabs, USA) and sequenced using the Hiseq 2500 platform (Illumina, USA). We removed low-quality sequences, which are those with over 50% bases having quality values of *Q* < 19 or those with over 5% bases being ‘N.’ We obtained a total of 49,157,518 reads as clean data for further analysis.

### 2.2. Genome assembly and annotation

We used NOVOPlasty (v2.7.2) (Dierckxsens et al. 2017) to perform de novo genome assembly from the clean data. Bowtie2 (v2. 0.1) (Langmead et al. 2009) was used to ensure the assembly's correctness by mapping all clean reads to the assembled genome sequences. We used CPGAVAS2 (Shi et al. 2019) to annotate the genome. We used Apollo (Misra and Harris 2006) to edit the annotations with problems manually. The simple sequence repeats (SSRs) were identified using the CPGAVAS2 web server by calling MISA (Beier et al. 2017), including mono-, di-, tri-, tetra-, penta-, and hexanucleotides with the minimum numbers were 10, 5, 4, 3, 3, and 3, respectively. Additionally, tandem repeats were detected with the Tandem Repeats Finder program (v4.07b). REPuter (Kurtz et al. 2001) was used to calculate palindromic repeats, forward repeats, reverse repeats, and complementary repeats with the settings: Hamming Distance was three, and Minimal Repeat Size was 30 bp.

### 2.3. Analysis of comparative genomics and divergence hotspots

The plastomes of ten *Photinia* species, including other nine published plastomes: *P. serratifolia* (NC_045331.1), *P. integrifolia* (NC_045344.1)*, P. beckii* (NC_045353.1), *P. lanuginosa* (NC_045354.1), *P. prionophylla* (NC_045355.1), *P. lochengensis* (NC_045352.1)*, P. glabra* (MK920277.1), *P. prunifolia* (MK920279.1), and *P. taishunensis* (MK920278.1) were compared by using shuffle-LAGAN mode in mVISTA to identify interspecific variations (Thiel et al. 2003; Frazer et al. 2004). We conducted a sliding window analysis using DnaSP (v6.0) to calculate the nucleotide polymorphism (Pi) among the ten species. Lastly, IRscope (Amiryousefi et al. 2018) was used for visualizing the IR boundaries of the plastomes.

### 2.4. Phylogenetic analysis

The plastome sequences of 24 species in the family Rosaceae, including two outgroup species (*Rosa rugosa* and *Sanguisorba officinalis*), were downloaded from GenBank (Supplemental Table S1). The complete plastome sequences and 75 common protein sequences among the 25 species were aligned by using CLUSTALW2 (v2.0.1) (Thompson et al. 2002), respectively. These proteins include ACCD, ATPA, ATPB, ATPE, ATPF, ATPH, ATPI, CCSA, CEMA, CLPP, MATK, NDHA, NDHB, NDHC, NDHD, NDHE, NDHF, NDHG, NDHH, NDHI, NDHJ, NDHK, PETA, PETB, PETD, PETG, PETL, PETN, PSAA, PSAB, PSAI, PSAJ, PSBA, PSBB, PSBC, PSBD, PSBE, PSBF, PSBH, PSBJ, PSBK, PSBM, PSBN, PSBT, PSBZ, RBCL, RPL14, RPL16, RPL2, RPL20, RPL22, RPL23, RPL32, RPL33, RPL36,RPOA, RPOB, RPOC1, RPOC2, RPS11, RPS12, RPS14, RPS15, RPS16, RPS18, RPS19, RPS2, RPS3, RPS4, RPS7, RPS8, YCF1, YCF2, YCF3 and YCF4. These aligned sequences were used to construct the phylogenetic trees by using the Maximum Likelihood method (ML) implemented in RaxML (v8.2.4) (Stamatakis 2014). The parameters were ‘raxmlHPC-PTHREADS-SSE3 -f a -N 1000 -m PROTGAMMALGX/GTRGAMMA -x 551314260 -p 551314260’. The bootstrap analysis were performed with 1000 replicates.

## 3. Results

### 3.1. Basic features of the plastomes

The plastomes of *Photinia* are characterized by a typical circular DNA molecule with a total length ranged from 159,230 bp (*P. beckii*) to 160,346 bp (*P. davidsoniae*). The overall GC content ranged from 36.42% to 36.66%. These plastomes have a conservative quartile structure, comprising a large single-copy (87,434–88,302 bp) region, a small single-copy (19,217–19,361 bp) region, and a pair of inverted repeat (26,280–26,436 bp) regions (Table 1). The GC content of IR regions is higher than that of SSC regions and LSC regions in all ten *Photinia* species.

**Table 1.** Basic features of the plastomes of ten *Photinia* species.

Species	Accession number	Length (bp)				GC Content (%)				Number of gene
		Total	LSC	SSC	IR	Total	LSC	SSC	IR	
*P. davidsoniae*	MT230547.1	160346	88302	19278	26383	36.42	34.03	30.33	42.66	131
*P. serratifolia*	NC_045331.1	160254	88210	19278	26383	36.44	34.05	30.34	42.66	131
*P. integrifolia*	NC_045344.1	159654	87563	19325	26383	36.53	34.26	30.18	42.63	131
*P. beckii*	NC_045353.1	159230	87434	19236	26280	36.66	34.4	30.39	42.66	131
*P. lanuginosa*	NC_045354.1	160184	88160	19258	26383	36.45	34.06	30.36	42.66	131
*P. prionophylla*	NC_045355.1	160333	88180	19281	26436	36.43	34.06	30.33	42.6	131
*P. lochengensis*	NC_045352.1	160201	88046	19361	26397	36.49	34.16	30.27	42.66	131
*P. glabra*	MK920277.1	159571	87786	19217	26284	36.54	34.22	30.31	42.7	131
*P. prunifolia*	MK920279.1	159757	87689	19238	26415	36.55	34.25	30.37	42.62	131
*P. taishunensis*	MK920278.1	159572	87765	19239	26284	36.54	34.23	30.29	42.7	131

### 3.2. Genome annotation

**Figure 1.**
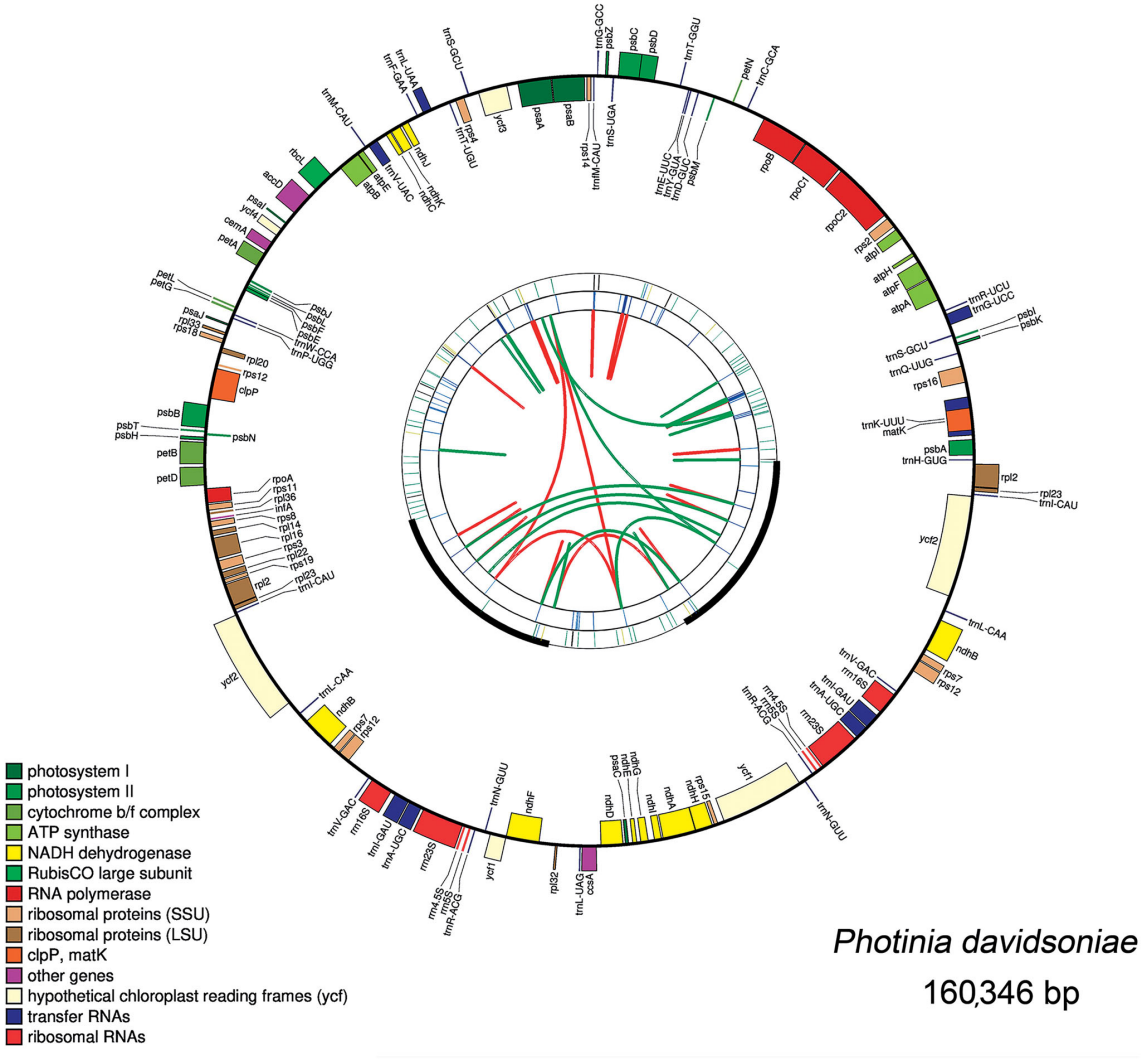
Graphic representation of features identified in *P. davidsoniae* plastome. The map was created with CPGAVAS2. Counted from the center, the first circle shows the forward and reverse repeats. They are connected with red and green arcs, respectively. The second and third circles indicate the locations of the tandem repeats and microsatellite sequences represented with short bars. The fourth circle shows the positions of plastome genes. The genes are colored base on their functional categories.

The genome structures of ten plastomes are highly conserved. Using the plastome of *P. davidsoniae* as an example, it contains 131 unique genes. Among them, 79 are protein-coding genes, four are rRNA genes, and 30 are tRNA genes (Figure 1 and Table 2). The total lengths of the protein-coding genes, rRNA genes, and tRNA genes are 77,832 bp, 9048 bp, and 2739 bp, accounting for 48.54%, 5.64%, and 1.71% of the complete plastome sequences, respectively. Introns play a significant role in selective gene splicing (Plangger et al. 2019). Among the 113 unique genes, two (*ycf*3 and *clp*P) contained two introns and 13 contained one intron, including eight protein-coding genes (*rps*16*, atp*F*, rpo*C1*, pet*B*, rpl*22*, rpl*2*, ndh*B*, ndh*A) and five tRNA genes (*trn*K-UUU*, trn*S-CGA*, trn*L-UAA*, trn*E-UUC*, trn*A-UGC) (Table S2). We identified six protein-coding genes, four rRNAs genes, and seven tRNA genes duplicated in the IR regions. Three genes have been found to span the IR and single-copy regions, namely *rps*19, *ndh*F, and *ycf*1. Their structures are described in the section describing the contraction/expansion of the IR regions.

**Table 2.** Gene contents of the plastomes of *Photinia* species.

Category of genes	Group of genes	Names of genes
rRNA	rRNA genes	*rrn*23S(×2), *rrn*16S(×2), *rrn*5S(×2), *rrn*4.5S(×2)
tRNA	tRNA genes	*trn*A-UGC, *trn*C-GCA, *trn*D-GUC, *trn*E-UUC, *trn*F-GAA, *trn*fM-CAU, *trn*G-GCC, *trn*G-UCC, *trn*H-GUG, *trn*I-CAU, *trn*I-CAU, *trn*I-GAU, *trn*I-GAU, *trn*K-UUU, *trn*L-CAA, *trn*L-CAA, *trn*L-UAA, *trn*L-UAG, *trn*M-CAU, *trn*N-GUU, *trn*N-GUU, *trn*P-UGG, *trn*Q-UUG, *trn*R-ACG, *trn*R-ACG, *trn*R-UCU, *trn*S-GCU, *trn*S-GCU, *trn*S-UGA, *trn*T-GGU, *trn*T-UGU, *trn*V-GAC, *trn*V-GAC, *trn*V-UAC, *trn*W-CCA, *trn*Y-GUA
Self-replication	The small subunit of the ribosome	*rps*11, *rps*12(×2), *rps*14, *rps*15, *rps*16, *rps*18, *rps*19, *rps*2, *rps*3, *rps*4, *rps*7(×2), *rps*8
	Large subunit of ribosome	*rpl*14, *rpl*16, *rpl*2(×2), *rpl*20, *rpl*22, *rpl*23(×2), *rpl*32, *rpl*33, *rpl*36
	DNA dependent RNA polymerase	*rpo*C1, *rpo*C2, *rpo*B, *rpo*A
Photosynthesis	Subunits of NADH-dehydrogenase	*ndh*A, *ndh*B(×2), *ndh*C, *ndh*D, *ndh*E, *ndh*F, *ndh*G, *ndh*H, *ndh*I, *ndh*J, *ndh*K
	Subunits of photosystem I	*psa*I, *psa*C, *psa*B, *psa*A, *psa*J
	Subunits of photosystem II	*psb*A, *psb*B, *psb*C, *psb*D, *psb*E, *psb*F, *psb*H, *psb*I, *psb*J, *psb*K, *psb*L, *psb*M, *psb*N, *psb*T, *psb*Z
	Subunits of cytochrome b/f complex	*pet*N, *pet*A, *pet*D, *pet*G, *pet*B, *pet*L
	Subunits of ATP synthase	*atp*I, *atp*E, *atp*A, *atp*B, *atp*H, *atp*F
	Large subunit of rubisco	*rbc*L
Other genes	Protease	*clp*P
	Envelope membrane protein	*cem*A
	Subunit of Acetyl-CoA-carboxylase	*acc*D
	c-type cytochrome synthesis gene Maturase	*ccs*A *mat*K
Genes of unknown functions Open Reading		*ycf*1(×2), *ycf*2(×2), *ycf*3, *ycf*4

*Note*. The ‘(x2)’ symbol after the gene name indicates that the genes locate on the IR regions and thus have two copies.

### 3.3. Repeats analysis

**Figure 2.**
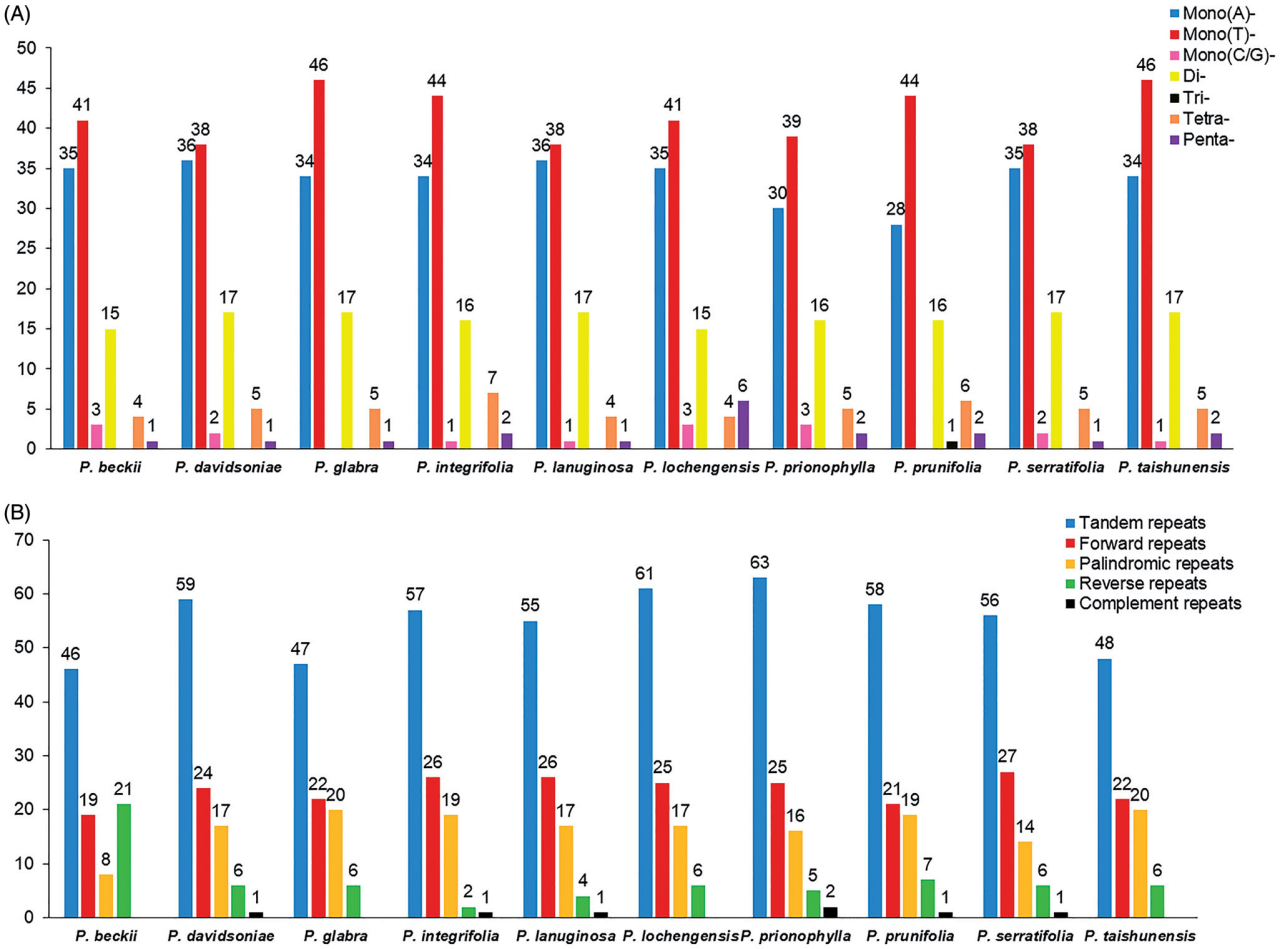
Comparison of the Repeats in the plastomes of *Photinia* species. (A) Types and numbers of SSRs detected in the plastomes of ten *Photinia* species; (B) Types and numbers of tandem repeats and dispersed repeats detected in the plastomes of ten *Photinia* species.

In this study, the numbers of SSRs ranged from 95 (*P. prionophylla*) to 105 (*P. taishunensis*). And we detected 1001 SSR loci in the ten plastome sequences (Figure 2A). It is worth noting that most of the repeating units in *Photinia* plastomes were A/T repeats, resulting in high A/T content in these plastomes. Besides, trinucleotide repeats are rare, and we only observed one in *P. prunifolia*.

Moreover, we detected 550 tandem repeats in the ten cp genomes using the similarity cutoff of 90% (Figure 2B). The numbers of tandem repeats ranged from 46 (*P. beckii*) to 63 (*P. prionophylla*). In contrast, the length of tandem repeats is mostly <= 20 bp (data are not shown). Besides, 237 forward repeats, 167 palindromic repeats, 69 reverse repeats, and seven complementary repeats were detected. Moreover, we found that *P. beckii* had the largest number of reverse repeats and the least number of palindromic repeats, different from the other nine species (Figure 2B).

### 3.4. Contraction and expansion of the IR regions

**Figure 3.**
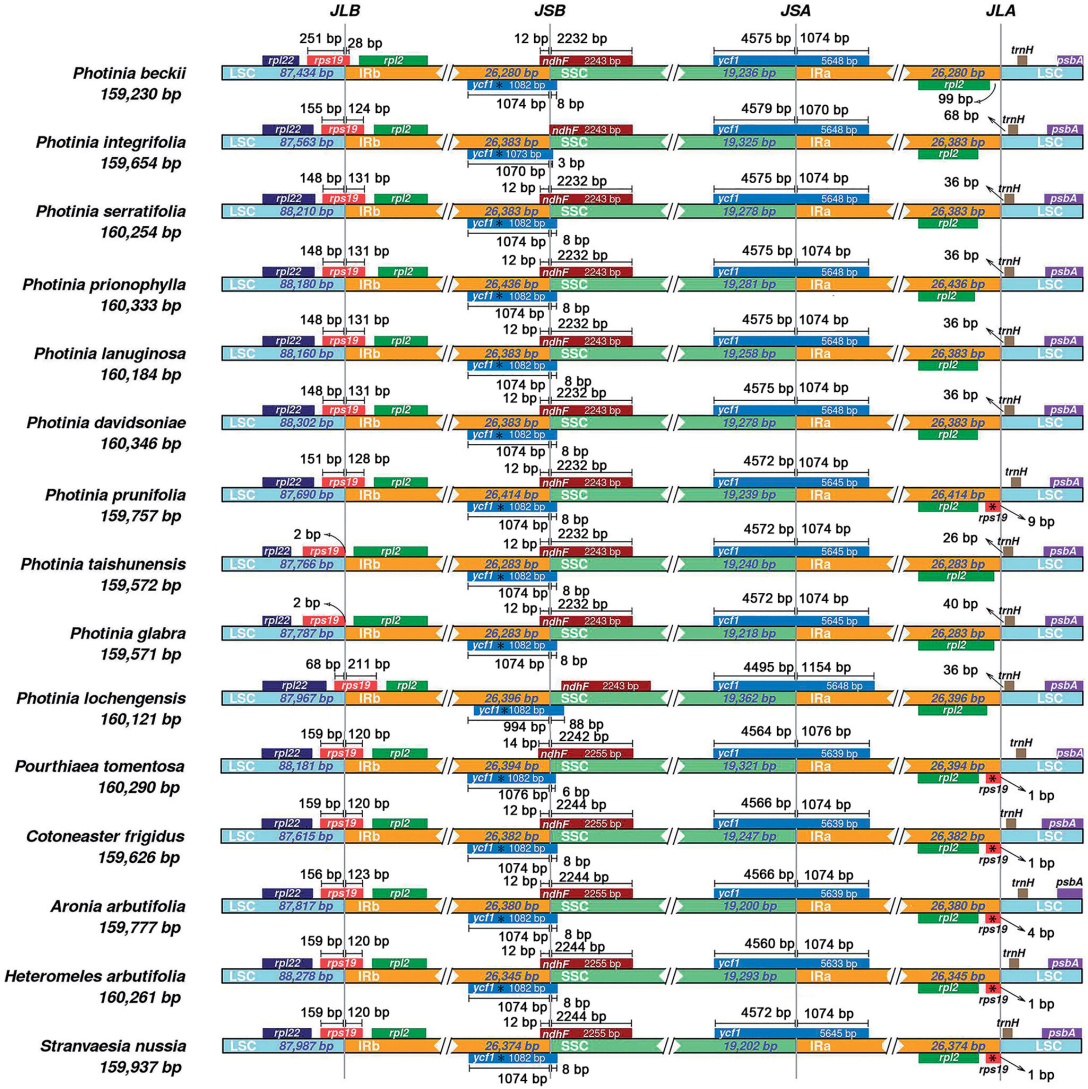
Comparison of LSC, SSC, and IR border regions of species from *Photinia* and related genera. The genes around the borders are shown above or below the mainline. The JLB, JSB, JSA, and JLA represent junction sites of LSC/IRb, IRb/SSC, SSC/IRa, and IRa/LSC, respectively. ‘*’: pseudogene.

With the evolution of plastomes, the IR regions have expanded and contracted, and some genes have the opportunity to access the IR regions or single-copy regions (Wang et al. 2018). The IR regions can undergo contraction and expansion, which are considered the main reason for the different lengths of plastomes in angiosperms. We compared the IR and SC boundaries of ten *Photinia* species and five related genera species (Figure 3). Three genes, *rps*19, *ndh*F, and *ycf*1, were found to span the borders. Most *rps*19 sequence is in the LSC region, and only a small fragment is in the IRb region. The length of the small fragment varies significantly. For example, it is 2 bp for *P. taishunensis* and *P. glabra*, 28 bp for *P. beckii*. In contrast, it is over 100 bp in other species.

For gene *ndh*F, they are located in the IRb/SSC border and overlapping with the first copy of *the ycf*1 gene. The large fragment of *ndh*F is in the SSC region; the small segment of *ndh*F is in the IRb region. The exception is that the *ndh*F genes for *P. integrifolia* and *P. lochengensis* are completely included in the SSC regions. For gene *ycf*1, there have two copies, which span the junctions of IRb/SSC and SSC/IRa. The length of the fragments in the IRa and IRb regions are similar, about 1000 bp.

### 3.5. Genome divergence and hypervariable regions

**Figure 4.**
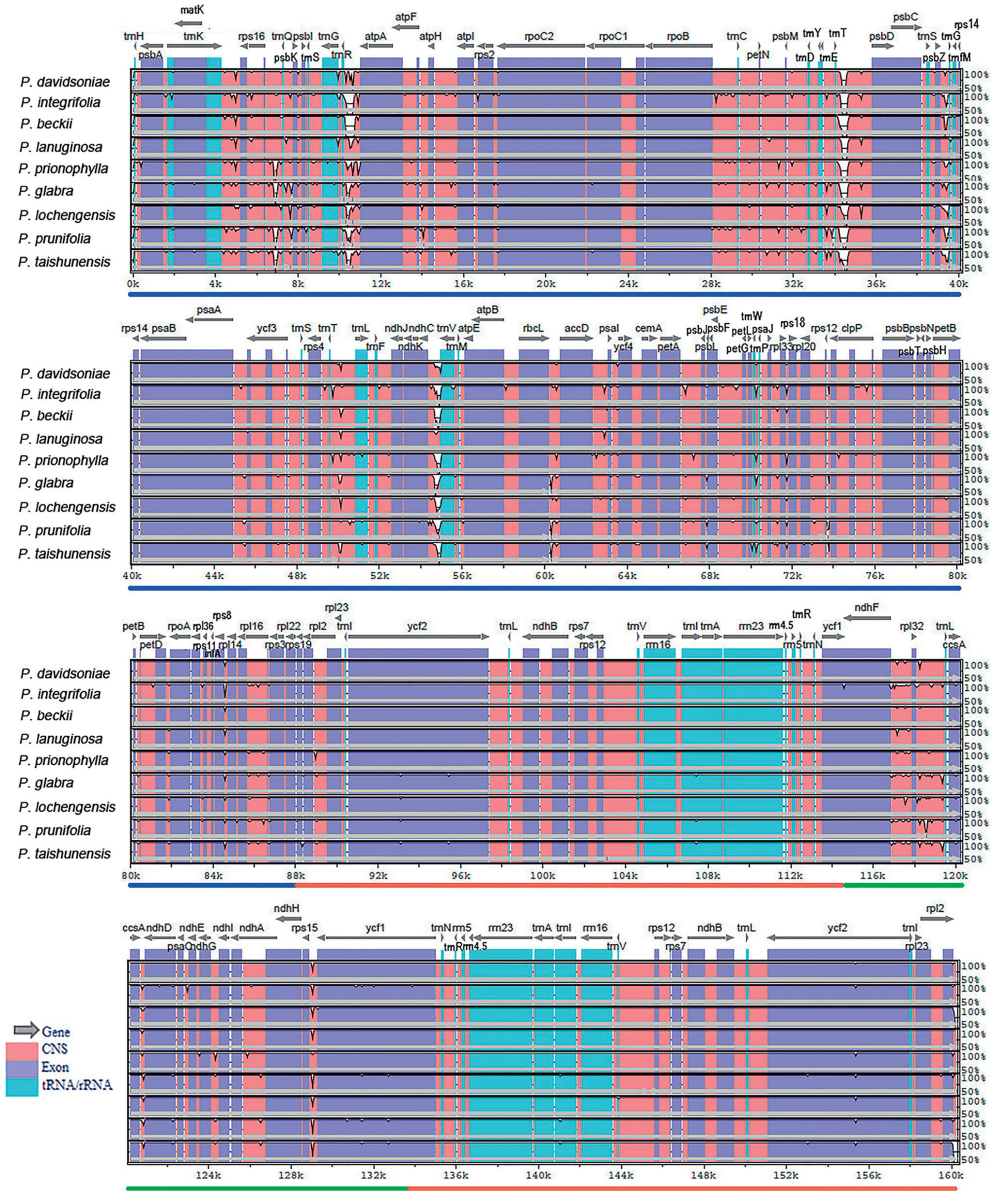
Comparison of the cp genomes in *Photinia* species by using mVISTA. The gray arrows on the top of the alignment represent genes. The pink regions are ‘Conserved Non-Coding Sequences’ (CNS), the dark blue regions are exons, and the light-blue regions are tRNA or rRNA. The percentages (50% and 100%) are the similarity among these sequences. Gray arrows on the top of the aligned sequences represent genes and their orientation. The colored lines under each window represent different regions of the plastomes: the blue line refers to the LSC region, the red line refers to the IR regions, and the green line refers to the SSC region.

**Figure 5.**
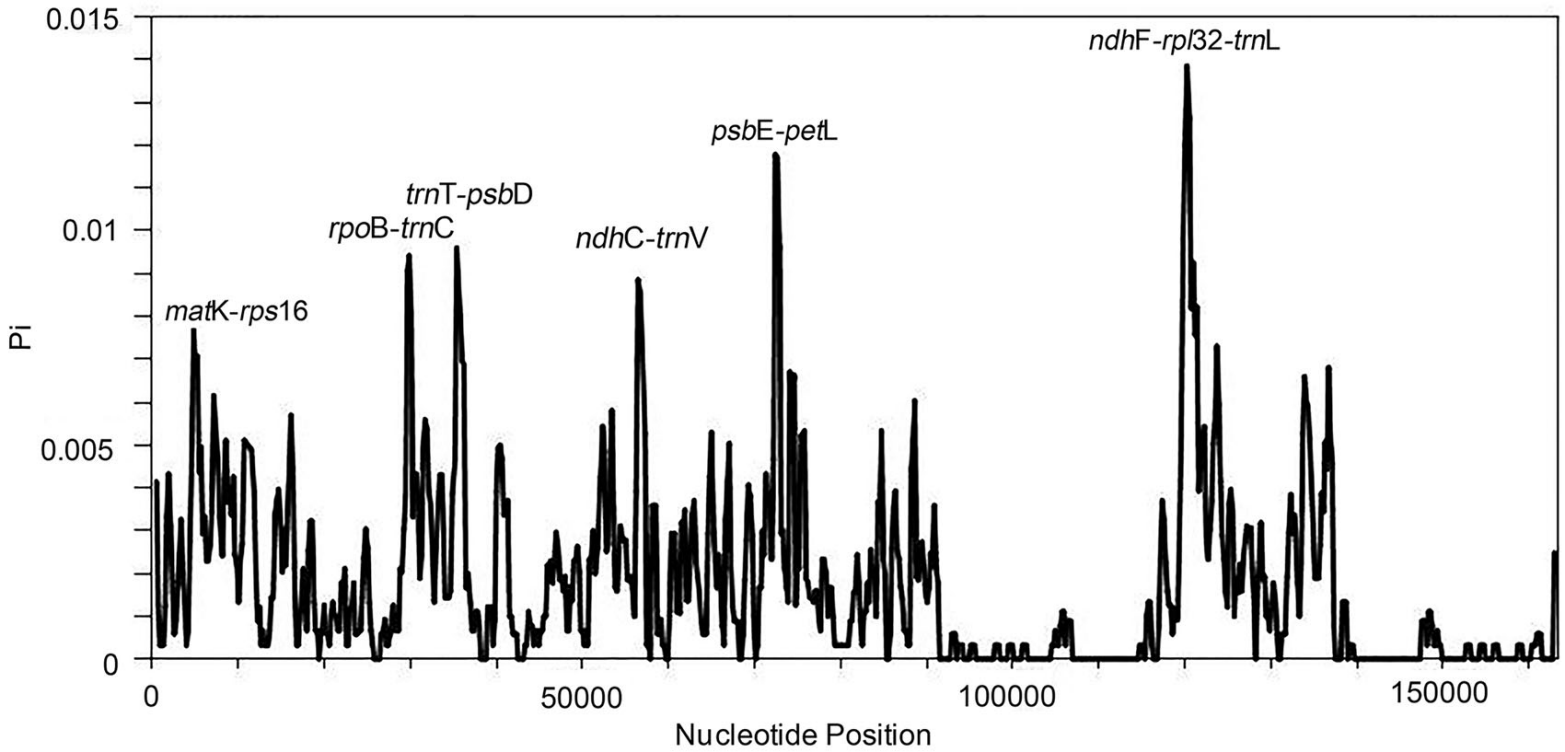
Hypervariable regions in *Photinia* species. We used a sliding window to analyze the sequence polymorphism among the plastomes of ten *Photinia* species. The sliding window has a length of 600 bp and a step size of 200 bp. The X-axis represents the position of nucleotide; Y-axis represents nucleotide polymorphism of each window.

To evaluate the genomic divergence, we analyzed ten plastomes using mVISTA. The LSC and SSC regions showed a high divergence level than the IR regions, particularly for the non-coding regions. Three non-coding regions showed significant divergence: *trn*R-*atp*A, *trn*T*-psb*D, and *ndh*C*-trn*V (Figure 4). Overall, most regions of the analyzed sequences showed a high degree of similarity. To quantify the levels of DNA polymorphism, the ten plastomes were analyzed by using DnaSP (v6.0) and we detected six hypervariable regions: *mat*K-*rps*16 (0.00767), *rpo*B*-trn*C (0.00941), *trn*T*-psb*D (0.00959), *ndh*C*-trn*V (0.00885), *psb*E-*pet*L (0.01178), and *ndh*F*-rpl*32-*trn*L (0.01385). The Pi values are listed in the parentheses. All of them are intergenic regions (Figure 5). These hypervariable regions could be used as potential molecular markers.

### 3.6. Phylogenetic analysis using plastomes data

**Figure 6.**
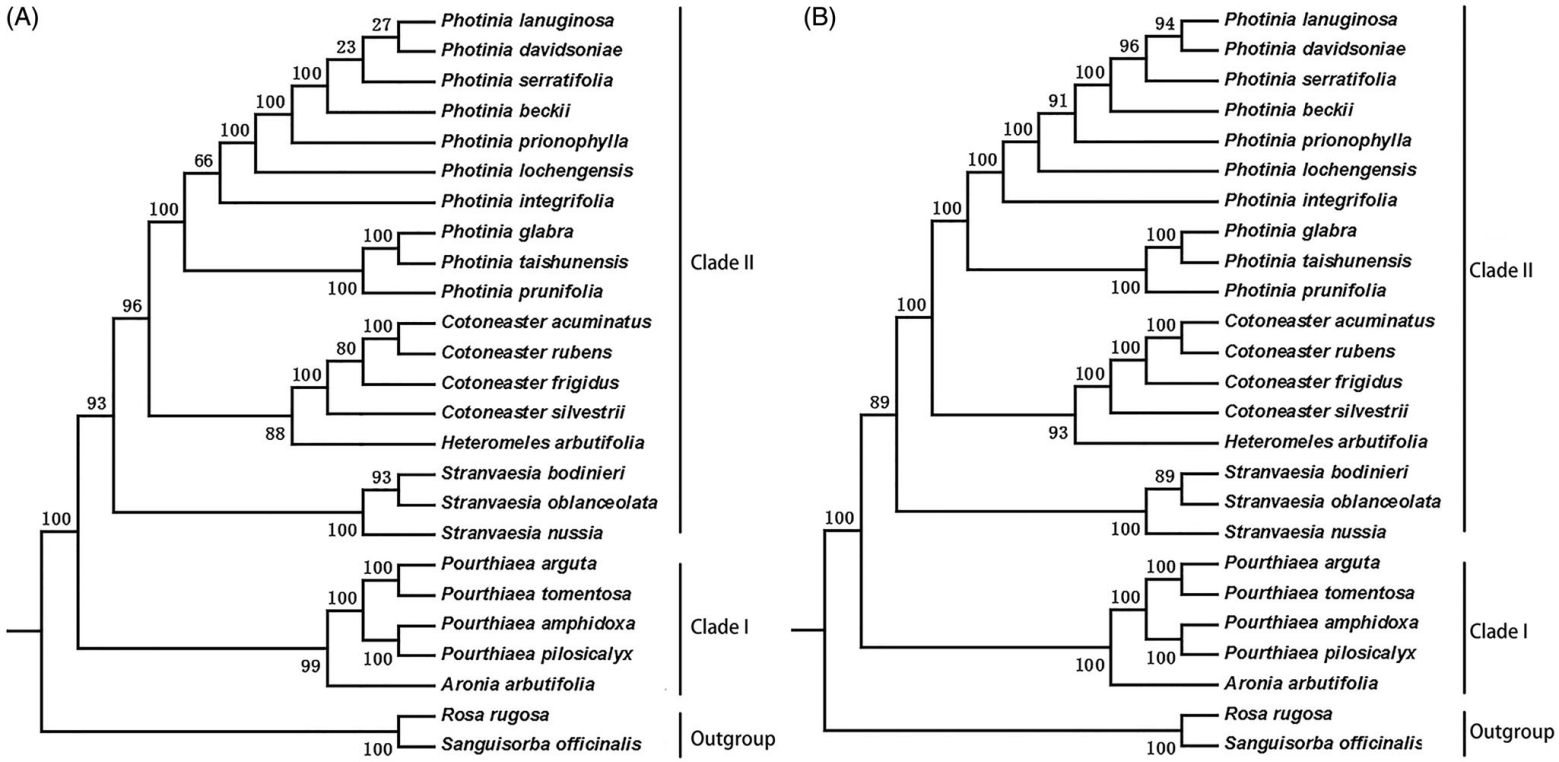
Phylogenetic relationships of species from *Photinia* and related genera inferred using the Maximum likelihood (ML) method. A. The phylogenetic tree was constructed was constructed using the complete nucleotide sequences of the using the 75 common protein sequences among the 25 cp genomes. B. The phylogenetic tree 25 cp genomes. Two taxa, namely, *R. rugosa* and *S. officinalis*, were used as outgroups. Bootstrap values were calculated from 1000 replicates.

Here, we use two data sets to determine the phylogenetic relationship: the common protein-coding sequences (Figure 6A) and the complete plastome sequences (Figure 6B). In our phylogenetic trees, it is evident that there are two main clades and then further divided into different subclades. Clade I included *Pourthiaea* and *Aronia*, and clade II contained four genera: the *Photinia*, *Heteromeles*, *Cotoneaster*, and *Stranvaesia*. Our data showed that *P. davidsoniae* is most closely related to *P. lanuginosa*. Both were most closely related to *P. serratifolia*. The two phylogenetic trees have similar topologies. However, the branches from the tree constructed with the complete chloroplast genome sequences have higher bootstrap support values. It is possible that the protein sequences are highly conserved and thus do not have sufficient informative sites to determine the relationships among these species.

## 4. Discussion

In this study, we sequenced the plastome of *P. davidsoniae* to understand its phylogenetic relationship with other congeneric species and also carried out a detailed comparative analysis of ten plastomes from *Photinia*.

The plastomes were found to be highly conserved from different aspects. For example, these plastomes have identical numbers of protein-coding genes, rRNA genes, and tRNA genes. There is no rearrangement among the plastomes, consistent with those described for most other genera in angiosperms (Raman et al. 2017). Furthermore, our phylogenetic results are consistent with the earlier investigation (Shi et al. 2019; Liu, Liu, et al. 2019). And the results show that the whole plastome sequences are more reliable in phylogenetic and evolutionary studies as a super barcode (Zhang, Zhang, et al. 2019).

Nevertheless, we have identified minor variations among these plastomes. Firstly, there are differences in the number of repeats detected in different species, including SSRs, tandem repeats, and dispersed repeats. SSRs exhibited high polymorphism in *Photinia* species, which have provided a large amount of information for molecular markers (Wang et al. 2017). Previous research reported that these short dispersed repeats ranged from 30 to 40 bp are essential for promoting plastomes rearrangements. Whether these repeats have caused the rearrangement of the cp genomes of *Photinia* species is an interesting question.

Secondly, changes were observed in the IR boundary region of these plastomes. Although these changes are subtle, two genes, *rps*19 and *ndh*F, exhibited significantly dynamic changes at LSC/IRb and IRb/SSC. For gene *rps*19, it didn’t span the LSC/IRb border in two species (*P. taishunensis* and *P. glabra*). Similarly, *ndh*F genes did not span the IRb/SSC boundary in some species (*P. integrifolia* and *P. lochengensis*). However, both genes span the boundaries in most cases. It is not clear whether this dynamic boundary change has any effect on these genes’ transcription.

Lastly, there were high nucleotide polymorphisms in the non-coding region of the plastomes. Some intergenic regions are potentially hypervariable regions for the development of molecular markers. We recommend six hypervariable regions, *mat*K-*rps*16 (0.00767), *rpo*B*-trn*C (0.00941), *trn*T*-psb*D (0.00959), *ndh*C*-trn*V (0.00885), *psb*E-*pet*L (0.01178), and *ndh*F*-rpl*32-*trn*L (0.01385), as potential molecular markers. These markers have important applications for rapid interspecific identification of *Photinia* taxa.

We learned several lessons from these studies. Firstly, for closely related species, the non-coding regions might provide useful information to understand the current evolutionary processes. Secondly, one needs to incorporate additional information from the nuclear genomes and mitochondrial genomes for overall phylogenetic and evolutionary analysis. Unfortunately, we could not retrieve the raw sequence data for these analyses. For the future, more genome sequencing is needed to further explore these issues.

In summary, the results reported here could provide valuable information for genetic diversity, phylogenetic evolution, and taxonomy studies of the genus *Photinia*.

## Data Availability

The sample has been deposited in the herbarium of the Institute of Medicinal Plant Development in Beijing, China, with the accession number: implad201808128. The genome sequence data that support the findings of this study are openly available in GenBank of NCBI at (https://www.ncbi.nlm.nih.gov/) under the accession no. MT230547. The associated BioProject, Bio-Sample, and SRA numbers are PRJNA688534, SAMN17180600, and SRR13325884, respectively.
